# Metabolic and endocrine profiles and reproductive parameters in dairy cows under grazing conditions: effect of polymorphisms in somatotropic axis genes

**DOI:** 10.1186/1751-0147-53-35

**Published:** 2011-06-02

**Authors:** Gretel Ruprechter, Mariana Carriquiry, Juan Manuel Ramos, Isabel Pereira, Meikle Ana

**Affiliations:** 1 Faculty of Veterinary Medicine and Agronomy Sciences, University of Uruguay, Montevideo, Uruguay; 2 University of the Enterprise, Montevido, Uruguay

## Abstract

**Background:**

The present study hypothesized that GH-AluI and IGF-I-SnabI polymorphisms do change the metabolic/endocrine profiles in Holstein cows during the transition period, which in turn are associated with productive and reproductive parameters.

**Methods:**

Holstein cows (Farm 1, primiparous cows, n = 110, and Farm 2, multiparous cows, n = 76) under grazing conditions were selected and GH and IGF-I genotypes were determined. Blood samples for metabolic/endocrine determinations were taken during the transition period and early lactation in both farms. Data was analyzed by farm using a repeated measures analyses including GH and IGF-I genotypes, days and interactions as fixed effects, sire and cow as random effects and calving date as covariate.

**Results and Discussion:**

Frequencies of GH and IGF-I alleles were L:0.84, V:0.16 and A:0.60, B:0.40, respectively. The GH genotype was not associated with productive or reproductive variables, but interaction with days affected FCM yield in multiparous (farm 2) cows (LL yielded more than LV cows) in early lactation. The GH genotype affected NEFA and IGF-I concentrations in farm 1 (LV had higher NEFA and lower IGF-I than LL cows) suggesting a better energy status of LL cows.

There was no effect of IGF-I genotype on productive variables, but a trend was found for FCM in farm 2 (AB cows yielded more than AA cows). IGF-I genotype affected calving first service interval in farm 1, and the interaction with days tended to affect FCM yield (AB cows had a shorter interval and yielded more FCM than BB cows). IGF-I genotype affected BHB, NEFA, and insulin concentrations in farm 1: primiparous BB cows had lower NEFA and BHB and higher insulin concentrations. In farm 2, there was no effect of IGF-I genotype, but there was an interaction with days on IGF-I concentration, suggesting a greater uncoupling somatropic axis in AB and BB than AA cows, being in accordance with greater FCM yield in AB cows.

**Conclusion:**

The GH and IGF-I genotypes had no substantial effect on productive parameters, although IGF-I genotype affected calving-first service interval in primiparous cows. Besides, these genotypes may modify the endocrine/metabolic profiles of the transition dairy cow under grazing conditions.

## Background

Energy balance of dairy cows becomes negative (NEB) during the transition period due to increased nutrient requirements that typically exceed dietary intakes. With the onset of lactation, plasma levels of non-esterified fatty acids (NEFA) and B-hidroxybutyrate (BHB) increase markedly, according to the magnitude of adipose tissue mobilization, to provide additional energy for maintenance and milk production [1-3]. Growth hormone (GH) is known to be responsible for galactopoiesis and persistency of lactation [1,4], and the uncoupled somatotropic axis (GH-insulin-like growth factor I axis, IGF-I) mediates nutrient partitioning for lactogenesis in high producing dairy cows [5]. Concentrations of GH are usually increased during early postpartum and its metabolic effects are antagonistic to insulin by enhancing lipolysis in the adipose tissue and gluconeogenesis in the liver [1,6,7]. Thus, insulin resistance develops to help direct nutrients from insulin-sensitive tissues to the lactating mammary gland [1]. Indeed, genetically-selected dairy cows had increased GH and reduced IGF-I and insulin concentrations during early lactation [8]. Since IGF-I and insulin affect ovarian function, low concentrations of these hormones during the postpartum period are associated with prolonged acyclicity [9-13]. As GH has proven to play a key role on the regulation of metabolism and milk production by modulating the expression of many genes, including IGF-I [14,15], these two genes - GH and IGF-I - could be considered candidate gene markers for productive and reproductive traits.

A polymorphic site of the GH gene that results in an amino acid change at position 127 - leucine, (L) to valine, (V) - detected by AluI, has been linked to milk production traits [16]. However, research results have been controversial as several authors [17-20], reported increased production traits associated with the L allele, while others [21-23] determined a favorable effect of the V allele on production. In contrast, Yao et al. [24] were not able to prove any association between this polymorphism and production traits. Very few studies have been performed regarding the relationship between GH-AluI genotype and reproduction [25-27]. Lechnniak et al. [25] reported that homozygous VV beef bulls tended to present greater non-return rates suggesting a beneficial effect on reproduction whereas no effect of this polymorphism was found on number and diameter of oocytes collected [26]. Balogh et al. [27] did not find an effect of this polymorphism on days to first postpartum ovulation in dairy cows.

A polymorphic site in the first promoter region of the bovine IGF-I gene was found by Ge et al. [28]. This polymorphism was identified as a point mutation, T (allele A) to C (allele B) transition, also referred to SnaBI by the same author. Unlike the abundant reports found in relation to GH-AluI genotype, scarce reports exist regarding the relationship between milk production and the IGF-I-SnabI genotype. Siadkowska et al. [29] determined that Polish Holstein-Friesian cows carrying the AB genotype yielded more daily fat-corrected-milk (FCM) than those of AA and BB genotypes, while Hines et al. [30] found no association between IGF-I-SnabI genotype and production traits in Holstein cattle. In addition, the BB genotype has been associated with greater body weight at weaning in commercial beef lines of *Bos taurus *[31] and greater growth rates in Holstein-Friesian bulls [29]. We have not found reports of IGF-I polymorphism and bovine reproduction.

Few studies performed in different bovine breeds and physiological stages focused on the mechanism by which these GH or IGF-I genotypes affect metabolic and endocrine profiles [32,28,34]. Only one report on the mentioned GH and IGF-I polymorphisms in dairy cow during the transition period was found. Balogh et al. [33] could not demonstrate any effect of GH-AluI genotype on BHB, insulin, IGF-I, and leptin concentrations in one blood sample collected between 4 and 13 days postpartum in Holstein Friesian dairy cows.

The present study hypothesized that GH-AluI and IGF-I-SnabI genotypes do change the metabolic and endocrine profiles in Holstein cows during the transition period, which in turn may be associated with the productive and reproductive responses.

## Materials and methods

### Animals and experimental design

Holstein cows under grazing conditions from two commercial dairy herds in Uruguay were used. All procedures were carried out in accordance with regulations of the Animal Experimentation Committee (Veterinary Faculty, University of Uruguay, Uruguay). Blood samples collected by coccygeal venopunction into tubes Vacutainer^® ^(Becton Dickinson, NJ, USA) containing K3EDTA were used to determine GH and IGF-I genotypes. Preliminary data of milk production and composition according to these GH and IGF-I genotype has been published before [35].

#### Farm 1

Primiparous Holstein cows that calved between March and May were randomly selected (n = 110) from a 700-cow herd. All cows grazed a mixture of ryegrass (*Lolium multiflorum*) in the morning and alfalfa (*Medicago sativa*) in the afternoon and were supplemented with 12 kg dry matter (DM) of corn silage, 5 kg DM of high-moisture corn grain, and 2 kg DM sunflower meal. The diet offered had 17% crude protein and 1.7 Mcal/kg DM of net energy of lactation (NRC, 2001). Cows were milked twice daily and milk yield and composition (fat and protein) were measured once monthly until the end of lactation. Body condition score (BCS) was determined at -7 ± 4, and exactly at 30 and 60 days postpartum (dpp) using a 5- point scale [36]. At the same time, blood samples for metabolites and hormones analyses were collected by coccygeal venopunction into heparinized tubes from 94 cows, centrifuged at 3000 Xg for 20 min and plasma was stored frozen (-20°C) until further analysis. The breeding period consisted of 4 months from June to September. Oestrus was detected twice a day and cows were artificially inseminated (AI), 12 hours after heat detection by the same inseminator. Pregnancy diagnosis was performed by rectal palpation 45 days after AI.

#### Farm 2

Multiparous Holstein cows (n = 60) that calved between September and November were randomly selected from 450-cow herd; cows had 1 (L2, n = 36) or 2 (L3, n = 24) previous lactations. During the last month before calving, cows grazed on a native pasture and received 11 kg DM/cow/day of a diet composed of 7 kg DM of sorghum silage, 3 kg DM of sorghum grain, 1 kg DM of sunflower meal (36% crude protein) and 100 g of urea. After calving, cows received a commercial mineral supplement and were managed under a rotational grazing system with supplementary feed added to maintain a pasture forage availability of 1,200 kg DM and an estimated total intake of 18 kg DM/cow/day. The diet offered had 17% crude protein and 1.5 Mcal/kg DM of net energy of lactation (NRC, 2001). Cows were milked twice daily and milk yield and composition (fat and protein) were measured once weekly for the first month of lactation and afterwards monthly until the end of lactation. Cow BCS was determined every 15 days from -30 to 60 dpp as described for farm 1. Blood samples for metabolites and hormones analyses were collected from 29 cows as described in farm 1 every 15 days from -30 dpp to calving, and then once a week up to 60 dpp. The breeding period consisted in 3 months from September to November: during the first two months AI was used, insemination 12 hours after oestrus detection twice a day, and natural mating was used during the last month. Pregnancy diagnosis was performed as described in farm 1.

### Laboratory analysis

Genotyping of GH and IGF-I and hormone (insulin and IGF-I) analyses were performed at the Nuclear Techniques Laboratory (Veterinary Faculty, University of Uruguay, Uruguay), while metabolites analyses (NEFA and BHB) were performed at DILAVE Laboratory (Pando, Uruguay).

Extraction of DNA was performed according to Kawasaki [37] and DNA was stored frozen (-20°C) until further analysis. The GH-AluI genotype was determined by a polymerase chain reaction-restriction fragment length polymorphism (PCR-RFLP) according to Lucy et al. [18]. Primers designed to amplify a 428-bp sequence of the bovine GH gene, GH For.: 5'-CCGTGTCTATGAGAAGC-3' and GH Rev.: 5'-TTCTTGAGCAGCGCGT-3'were used. A Digestion of PCR product was performed with 6U of AluI (Fermentas Inc., MD, USA) restriction endonuclease. Fragments of DNA were resolved in a 2% agarose gel stained with ethidium bromide (EtBr) and fragments of either leucine (L; 265, 96, 51 and 16 bp) or valine (V; 265, 147 and 16 bp) alleles were visualized under UV light (Cleaver Scientific, England).

The IGF-I-SnabI genotype was determined by PCR-RFLP according to Ge et al, [28]. Primers designed to amplify a 249-bp sequence of the bovine IGF-I gene, IGF-I For.: 5`-ATTACAAAGCTGCCTGCCCC-3` and IGF-I Rev.: 5`-ACCTTACCCGTATGAAAGGAATATACGT-3`were used and PCR products digestion was performed with 5U of SnabI (Fementas Inc., MD, USA) restriction endonuclease. The DNA fragments were resolved in a 3% agarose gel stained with EtBr and fragments of either T (A) (223 and 26 bp) or C (B) (undigested, 249 bp) alleles were visualized under UV light (Cleaver Scientific, England).

Plasma insulin concentrations were determined by a ^125^I-Insulin RIA kit (Diagnostic Products Co., Los Angeles, California, USA). The assay sensitivity was 1.3 μIU/mL and the intra-assay and inter-assay coefficients of variation were less than 8.2 and 10.1% for control 1 (4.2 μIU/mL) and 9.4 and 11.3% for control 2 (12.6 μIU/mL), respectively. Plasma IGF-I concentrations were determined by the IGF1 RIACT (Cis Bio International, GIF SUR YVETTE CEDEX, France). The assay sensitivity was 16 ng/mL and the intra-assay coefficient of variation were 3.4 and 5.8% for control 1 (50.4 ng/mL) and 16 and 17% for control 2 (709 ng/mL), respectively.

Plasma NEFA and BHB concentrations were assayed by spectrophotometry using commercial kits: Kat. #FA 115 kit (Wako Chemicals, Richmond, VA, USA) and Kat. #RB 1007 (Randox Laboratories Ltd, Ardmore, UK), respectively. The intra-and inter-assay coefficient of variations for both metabolites were less than 7.3 and 9.7%, respectively.

### Statistical analyses

Data were analyzed in a complete randomized design by farm using the SAS program (Statistical Analysis System; SAS Institute Inc., Cary, NC, USA). Univariate analyses were performed on all variables to identify outliers and inconsistencies and to verify normality of residuals. Production traits and hormone and metabolite concentrations were analyzed by repeated measures using the MIXED procedure with days as the repeated effect and first-order autoregressive (for evenly spaced data) or spatial power law (for unevenly spaced data) as the covariance structure. The Kenward-Rogers procedure was used to adjust the denominator degree of freedom. The model included GH and IGF-I genotypes, dpp, and interactions as fixed effects, and sire and cow as random effects and calving date as covariate. Interactions remained in the model if P < 0.10. Pearson's linear correlation was estimated between predicted and observed data to evaluate model adjustment. Reproductive traits (calving-first service interval, number of services per conception and total pregnancy rate) were analyzed with a generalized linear model (GENMOD procedure) with a Poisson distribution and log transformation (calving-first service interval) or a binomial distribution and logit transformation (pregnancy rate). The model included the effect of GH and IGF-I genotypes as fixed effects and calving date as a covariate. Results are expressed as lsmeans (LSM) ± SE. For all results, means were considered to differ when P ≤ 0.05 and trends were identified when 0.05 < P < 0.10.

## Results

A χ2 test showed that allele frequency and genotypes of GH and IGF-I were in Hardy-Weinberg equilibrium (P = 0.97) and did not differ between farms (P > 0.28). GH allele frequencies were L (0.84) and V (0.16), while IGF-I allele distribution were A (0.60) and B (0.40). The number of cows for each genotype was LL (n = 122), LV (n = 51) and VV (n = 4) for GH genotypes and AA (n = 63), AB (n = 98) and BB (n = 25) for IGF-I genotypes. Due to the unequal distribution of GH genotypes in our study (dominance of the L allele and low frequency of V allele) we exclude VV genotype from further analysis.

### Productive and reproductive responses

**Figure 1:**
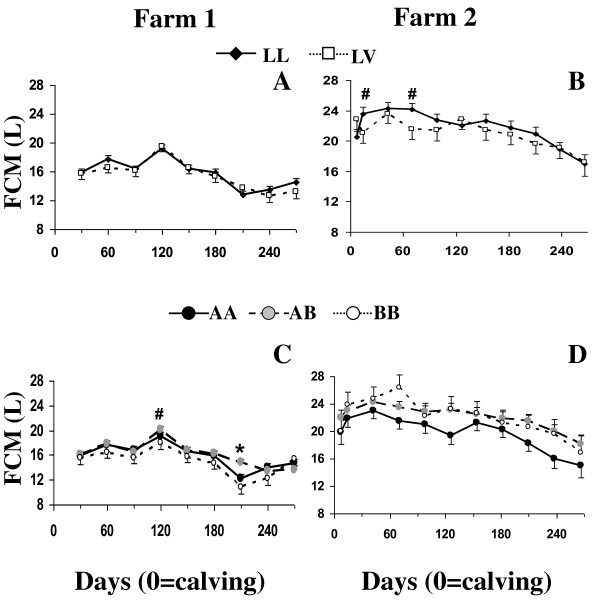
**Fat corrected milk yield for LL and LV genotypes (A, B) and AA, AB, and BB genotypes (C, D) of Holstein cows in Farm 1 (A, C) and Farm 2 (B, D)**. Asterisks denote differences at P < 0.05, while # denotes trends 0.05 < P < 0.10.

Correlations between predicted and observed values for all productive and reproductive variables were between 0.47 and 0.81. The GH genotype was not associated with productive variables in either of the farms (Tables 1 and 2, Figure 1A and 1B). While no effect of the interaction between GH genotype and dpp on productive variables was observed in farm 1 (primiparous cows), a trend was observed on 4%FCM yield (P = 0.07) in farm 2 (multiparous cows), as LL cows presented greater FCM yield than LV cows during early lactation (15 and 75 dpp, Figure 1B). The GH genotype had no effect on reproductive variables in none of the farms studied (Tables 1 and 2).

**Table 1:** F-tests of fixed effects included in the model for productive/reproductive parameters and metabolic/endocrine variables and BCS of Holstein cows under grazing conditions in two commercial farms.

	Farm 1			Farm 2		
	
	GH	IGF-I	dpp	GH	IGF-I	dpp
Milk (L)	0.43	0.25	< 0.01	0.41	0.18	< 0.01
FCM (L)	0.54	0.24	< 0.01	0.56	0.09	< 0.01
Total solids (kg)	0.50	0.23	< 0.01	0.44	0.13	< 0.01
Calving 1^st ^service (days)	0.26	< 0.01	-	0.23	0.17	-
Service/conception	0.74	0.68	-	0.45	0.90	-
Pregnancy rates	0.97	0.96	-	0.80	0.97	-
BCS	0.42	0.58	0.86	0.42	0.95	< 0.01
BHB (mmol/L)	0.31	0.01	0.09	0.86	0.77	< 0.01
NEFA (mmol/L)	0.01	< 0.01	0.06	0.77	0.44	< 0.01
Insulin (μUI/mL)	0.99	0.02	< 0.01	0.53	0.91	< 0.01
IGF-I (ng/mL)	0.09	0.34	< 0.01	0.85	0.95	< 0.01

Fixed effects are GH and IGF-I genotype and days post partum (dpp).

**Table 2:** Productive/reproductive parameters and metabolic/endocrine variables (LSM ± SE) for GH genotypes of Holstein cows in two commercial farms.

	Farm 1			Farm 2		
	GH genotype			GH genotype		
	
	LL	LV	SE	LL	LV	SE
Milk (L)	17.9	17.5	0.50	23.1	22.1	0.80
FCM (L)	15.9	15.5	0.45	21.6	21.0	0.91
Total solids (kg)	1.14	1.11	0.03	1.60	1.54	0.06
Calving 1^st ^service (days)	88	86	9	79	83	4
Service/conception	2.3	2.2	0.2	1.3	1.6	0.1
Pregnancy rates	80	78	7	72	64	10
NEFA (mmol/L)	0.39^**a**^	0.41^**b**^	0.02	0.34	0.33	0.03
BHB (mmol/L)	0.25	0.26	0.01	0.63	0.60	0.06
Insulin (μUI/mL)	3.5	3.5	0.23	2.60	2.81	0.38
IGF-I (ng/mL)	98.5^**x**^	79.4^**y**^	7.45	113.64	113.92	11.14
BCS	2.9	2.7	0.2	3.04	3.00	0.04

^ab ^Values marked with different letters are significantly different at P < 0.05

^xy ^Values marked with different letters are a trend at P < 0.10

The IGF-I genotype had no effect on the productive variables in farm 1 (Tables 1 and 3), but a trend for an effect of the interaction of IGF-I genotype and dpp was observed in 4%FCM yield (P = 0.09), as AB cows yielded more FCM than BB cows at 120 and 210 dpp (Figure 1C). In farm 2, IGF-I genotype tended (P = 0.09) to affect FCM yield (Table 1) as AB cows had greater FCM yield than AA cows (P = 0.03), while no differences were found between AB and BB cows. Fat-corrected-milk yield was numerically greater for BB than AA cows (21.9 vs. 19.7 ± 1.05 kg/d) but this difference did not reach significance (P = 0.16) (Table 3, Figure 1D).

**Table 3:** Productive/reproductive parameters and metabolic/endocrine variables (LSM ± SE) for IGF-I genotypes of Holstein cows in two commercial farms.

	Farm 1 IGF-I genotype					Farm 2 IGF-I genotype		
	
	AA	AB	BB	SM	AA	AB	BB	SM
Milk (L)	18.0	18.2	16.9	0.56	21.4	23.4	22.8	0.96
FCM (L)	15.9	16.2	15.0	0.51	19.7^**a**^	22.1^**b**^	21.9^**ab**^	1.05
Total solids (kg)	1.13	1.16	1.08	0.04	1.46^**a**^	1.62^**b**^	1.60^**ab**^	0.07
Calving 1^st ^service (days)	85^ab^	73^b^	103^a^	10	85	81	77	5
Service/conception	2.2	2.5	2.2	0.3	1.5	1.4	1.5	0.2
Pregnancy rates	80	82	74	8	68	69	66	10
NEFA (mmol/L)	0.42^**a**^	0.41^**a**^	0.37^**b**^	0.01	0.32	0.36	0.34	0.03
BHB (mmol/L)	0.27^**a**^	0.27^**a**^	0.24^**b**^	0.01	0.62	0.66	0.56	0.07
Insulin (μUI/mL)	2.91^**a**^	3.19^a^	4.18^**b**^	0.27	2.60	2.55	2.60	0.42
IGF-I (ng/mL)	85.8	97.5	94.8	7.45	117.8	112.9	115.9	12.2
BCS	2.7	2.9	2.7	0.2	3.00	3.02	3.03	0.05

^ab ^Values marked with different letters are significantly different at P < 0.05

The IGF-I genotype had a significant effect on calving-first service interval only in farm 1 (Table 1), as AB cows had a shorter interval than BB cows (Table 3). No other effect of IGF-I genotype was observed on reproductive variables (Table 3).

### Metabolic and endocrine profiles

**Figure 2:**
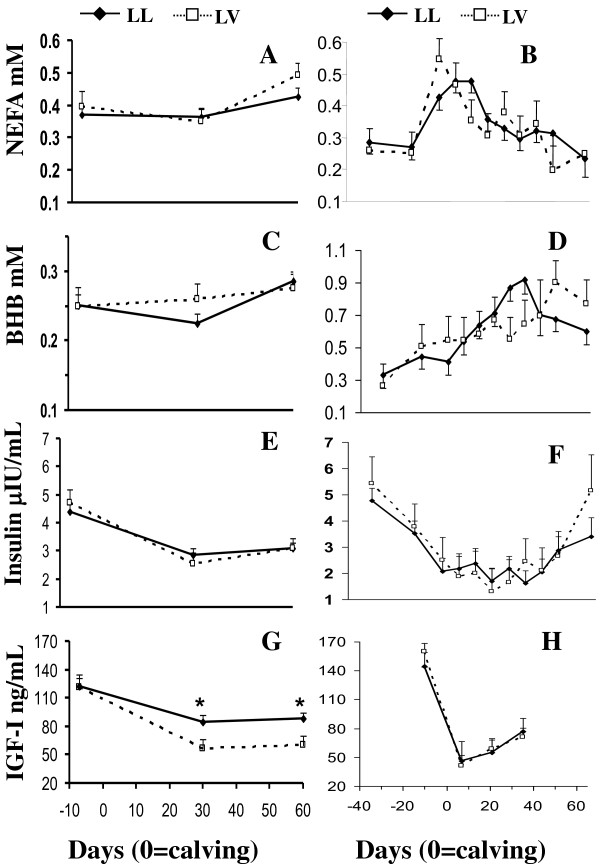
**Non-sterified fatty acids (NEFA, A, B), β-hydroxybutirate (BHB, C, D), insulin (E, F) and insulin like growth factor I (IGF-I, G, H) concentrations for LL and LV genotypes of Holstein cows in Farm 1 (A,C,E,G) and Farm 2 (B,D,F,H)**. Asterisks denote differences at P < 0.05.

In farm 1, the GH genotype affected or tended (P = 0.09) to affect plasma NEFA and IGF-I concentrations, respectively, but did not affect BCS or any other metabolic parameters (Table 1, Figure 2 A-H). Cows carrying LV genotype had greater plasma NEFA and tended to present lower IGF-I concentrations than LL cows (Table 2). Although plasma IGF-I concentrations decreased (P < 0.01) after calving in both genotypes, LV cows presented lower IGF-I concentrations at 30 and 60 dpp (P < 0.02) than LL cows (Figure 2G). In farm 2, GH genotype did not affect BCS or any of the endocrine/metabolic profiles (Table 1).

**Figure 3:**
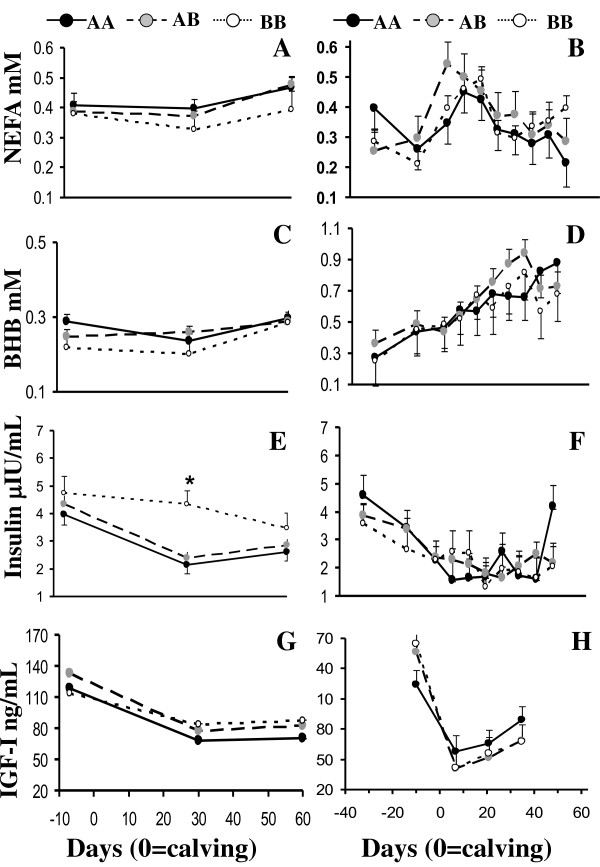
**Non-sterified fatty acids (NEFA, A, B), β-hydroxybutirate (BHB, C, D), insulin (E, F) and insulin like growth factor I (IGF-I, G, H) concentrations for AA, AB and BB genotypes of Holstein cows in Farm 1 (A,C,E,G) and Farm 2 (B,D,F,H)**. Asterisks denote differences at P < 0.05.

The IGF-I genotype affected BHB, NEFA, and insulin concentrations in farm 1 (Table 1) as BB cows presented lower plasma NEFA and BHB and greater insulin concentrations than AA and AB cows (Table 3, Figure 3 A, C, E). While insulin concentrations declined (P < 0.01) from -7 to 30 and 60 dpp for AA and AB cows, plasma insulin was maintained during the study in BB cows; being insulin concentrations at 30 dpp greater in BB than AA and AB cows (P < 0.01) (Figure 3 E). In farm 2, the interaction between IGF-I genotype and dpp tended (P = 0.06) to affect IGF-I concentrations as AA cows tended (P < 0.07) to present lower prepartum IGF-I concentrations than AB and BB cows, and although all cows presented a decline (P < 0.01) in IGF-I concentrations during the postpartum period, this decline was less pronounced in AA than AB and BB cows (Figure 3 H).

There was an effect of dpp on NEFA, BHB, insulin and IGF-I concentrations (Table 1). Metabolic and endocrine profiles were better characterized in farm 2; the concentrations of NEFA peaked around calving, and returned to basal levels at 30 dpp (Figure 2 B). The concentrations of BHB increased from -20 to 35 dpp, not returning to basal levels along the study (Figure 2 D). Insulin concentrations decreased from -30 dpp to calving, remained reduced until 50 dpp when insulin concentrations started to increase (Figure 2 F). Plasma IGF-I concentrations showed a sharp decrease at calving and increased thereafter without reaching prepartum levels at 35 dpp (Figure 2 H).

## Discussion

The GH and IGF-I allele frequencies in this study are in agreement with those reported previously in Holstein-Friesian for GH [18,19,27] and in Holstein for IGF-I [29-31].

There was no significant association between GH genotype and productive parameters (milk, 4%FCM and total solid yields) in accordance with Yao et al. [24] in Holstein bulls and Balogh et al. [27] in Holstein-Frisian cows. However, a trend was found for the interaction of GH genotype and dpp on FCM yield in farm 2 (multiparous cows), as LL cows produced more than LV cows during early lactation. Similarly, Shariflou et al. [19] suggested that the L allele appeared to have an additive effect on milk production only at the beginning of the lactation. Besides, Lucy et al. [18] reported that cows carrying LL genotype yielded more milk, fat, and protein than LV cows. No effect of the interaction between GH genotype and dpp was found in primiparous cows (farm 1), and this could be associated with the level of production and/or a differential role of GH genotype in growing animals. In contrast, Dybus et al. [20] determined an effect of GH genotype on milk, fat and protein yield in primiparous but not in multiparous cows, and they suggested that the observed differences could have resulted from another source of variation (e.g. effects of herd, sires) not considered in the study.

The IGF-I genotype tended to affect FCM yield in farm 2 (multiparous cows), and the interaction between IGF-I genotype and dpp tended to affect FCM yield in farm 1 (primiparous cows), as AB cows yielded more FCM than BB (farm 1) or AA (farm 2) cows. Similarly, Siadkowska et al. [29] reported that AB cows yielded more FCM than AA and BB cows. In contrast, Hines et al. [30] did not find any effect of IGF-I genotype on productive parameters. In our study, cows were under grazing conditions and were average producing cows (17 L/day in farm 1 and 22 L/day in farm 2). Previous studies that could not find any effect of the genotype on milk production stated that genotype differences might not be expressed at this level of production [38,27]. In addition to this, Chilibroste et al. [39] and Kolver and Muller [40] reported that DM intake is not enough to achieve the genetic potential on grazing milk production systems.

In our study there was no effect of GH genotype on reproductive parameters in none of the farms. Balogh et al. [27] found no effect of the GH genotype on the time of the first pospartum ovulation. Lechniak et al. [25] reported a tendency for greater non-return rates of VV beef bulls at 60 dpp and Lechniak et al. [26] found no effect of the GH genotype on oocyte number. No data as such has been found for the relationship between IGF-I genotype and reproduction in dairy cows. For IGF-I genotype there was a significant effect on calving-first service interval only in farm 1, as BB cows had a longer interval than AB cows.

We found only one report regarding the effects of GH genotype [33] and none of IGF-I genotype on metabolic and/or endocrine profiles in the transition dairy cow. Balogh et al. [33] did not found either an effect of GH genotype on plasma BHB, insulin, and IGF-I concentrations, but they performed only one postpartum determination (4 to 13 dpp). In the present study we have included pre and postpartum determinations which in our understanding, allowed a better comprehension of the metabolic endocrinology during the peripartum period.

In farm 1 (primiparous cows), NEFA and IGF-I concentrations were affected by GH genotype, as LL cows had lower NEFA and greater IGF-I concentrations than LV cows. Since NEFA and IGF-I are both indicators of the metabolic status [5], these data suggest that LL cows presented a better energy status than LV cows. It is supposed that bovine GH with Leu^127 ^stimulate the release of IGF-I more than other variants of bGH [41] which is consistent with the results found in the present study. In contrast, Schlee et al. [32] observed that Simmental LV bulls presented greater IGF-I concentrations. This differential metabolic/endocrine environment was not reflected on productive/reproductive traits, which could be due to the level of production of primiparous cows as discussed before and/or to the extra energy demands for growth in these cows. In farm 2 (multiparous cows) there was no effect of GH genotype on any of the metabolites and hormones studied. Although a reduced number of animals were included in this farm, there are more metabolic/endocrine time measurements which allowed a better metabolic description of the NEB. Indeed, NEFA and β-hydroxibutirate concentrations increased around calving reflecting fat mobilization as reported before [1-3]. As expected, insulin concentrations decreased around calving as has previously been observed [13]. This decrease in plasma insulin is a metabolic adaptation to cope with the energy demands of lactation as reported earlier [42,43], since low insulin concentrations favours gluconeogenesis and lipolysis [44] (e.g. homeorhetical effect). The decrease in IGF-I concentrations at calving confirmed the uncoupled somatotropic axis (GH-IGF-I), which mediates nutrient partitioning for lactogenesis [5]. We have no obvious explanation for the differential effect of GH genotype on metabolic/endocrine profiles found in primiparous cows (farm 1) vs. multiparous cows (farm 2), but as previously suggested it could be due to the differential role of GH during growth and development. Indeed, primiparous cows present greater insulin and IGF-I concentrations than multiparous cows [42].

Insulin-like growth factor-I genotype affected NEFA and BHB concentrations in farm 1 (primiparous cows); as BB cows had lower concentrations than AA and AB cows. In accordance to the better energy balance in BB cows reflected by these metabolites, these cows presented greater insulin concentrations at 30 dpp. Unexpectedly, IGF-I concentrations were not affected by IGF-I genotype. Ge et al. [28] and Maj et al. [34] reported lower or greater IGF-I blood concentrations in BB young Angus cattle or BB Holstein -Friesian young bulls and heifers, respectively. Since this polymorphism is located in the promoter region of the IGF-I gene, a variety of responses in gene expression may result depending on the physiological and/or nutritional status of the animal. This differential energy balance is not consistent with the calving first service interval, since it is known that cows in a better energy balance have also better reproductive performance [45], and in this study BB cows presented longer calving-first service interval than AB cows (103 vs 73 days, respectively). Unfortunately, we do not have the endocrine/metabolic profiles at the time of the initiation of the services which could clarify these contradictory results; indeed the endocrine system changes dynamically according to the nutritional and productive status. Moreover, BB cows had not only a reduced reproductive performance but they tended to yield less FCM at 120 and 210 dpp than AB cows. Staples and Thatcher [46] reported that cows with more DM intake, present not only greater milk production, but also better reproductive performance. In farm 2 (multiparous cows), there was no effect of IGF-I genotype on plasma NEFA, BHB, and insulin concentrations. On the other hand, IGF-I profiles suggest a greater uncoupling of the somatotropic axis in AB and BB cows than AA cows which is in accordance with the greater FCM yield of AB than AA cows.

## Conclusions

In summary, the GH - AluI and IGF-I - SnabI genotypes did not have a relevant effect on productive parameters, although the latter genotype affected calving-first service in primiparous cows. On the other hand, this study demonstrated that these genotypes do alter the endocrine and metabolic profiles of the transition dairy cow under grazing conditions.

## Competing interests

The authors declare that they have no competing interests.

## Authors' contributions

GR lead the experimental designs, carried out the hormone and the metabolites determinations and genotyping, and drafted the manuscript. IP and JM contributed with the experimental designs. MC contributed with the statistical analysis and helped together with AM on data interpretation and manuscript corrections. All authors read and approved the final manuscript.
